# *N*,*N*′-Bis(2-amino­benz­yl)ethane-1,2-diaminium dinitrate

**DOI:** 10.1107/S1600536813027475

**Published:** 2013-10-16

**Authors:** Luis Ángel Garza Rodríguez, Perla Elizondo Martínez, Sylvain Bernès, Blanca Nájera Martínez, Nancy Pérez Rodríguez

**Affiliations:** a Universidad Regiomontana A.C., 15 de Mayo 567 Pte., Monterrey, Nuevo León CP 64000, Mexico; b Universidad Autónoma de Nuevo León, UANL, Facultad de Ciencias Químicas, Av. Universidad S/N, Ciudad Universitaria, San Nicolás de los Garza, Nuevo León CP 66451, Mexico

## Abstract

In the title salt, C_16_H_24_N_4_^2+^·2NO_3_^−^, both the cation and anion are placed in general positions, although the cation displays non-crystallographic inversion symmetry, with the aliphatic chain extended in an all-*trans* conformation. The benzene rings are almost parallel, with a dihedral angle between their mean planes of 3.3 (6)°. The nitrate ions are placed in the vicinity of the protonated amine groups, forming efficient N—H⋯O inter-ion hydrogen bonds. Each nitrate ion in the asymmetric unit bridges two symmetry-related cations, forming an *R*_4_^4^(18) ring, a common motif in organic ammonium nitrate salts. This results in the formation of chains along [010] with alternating cations and anions. The neutral amine groups are involved in slightly weaker N—H⋯O hydrogen bonds with the nitrate O atoms, and there are also a number of C—H⋯O hydrogen bonds present. The resulting supra­molecular structure is based on a two-dimensional network extending in the *ab* plane.

## Related literature

For the structure of the free neutral amine, see: Rodríguez de Barbarín *et al.* (2007 ▶). For the *p*-toluene­sulfonate salt of the title cation, see: Garza Rodríguez *et al.* (2011 ▶). For related di­ammonium nitrate salts featuring 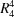
(18) motifs, see: Liu *et al.* (2007 ▶); Yang *et al.* (2007 ▶). For supra­molecular motifs nomenclature, see: Etter (1990 ▶). For the synthesis of the title salt, see: Garza Rodríguez (2010 ▶).
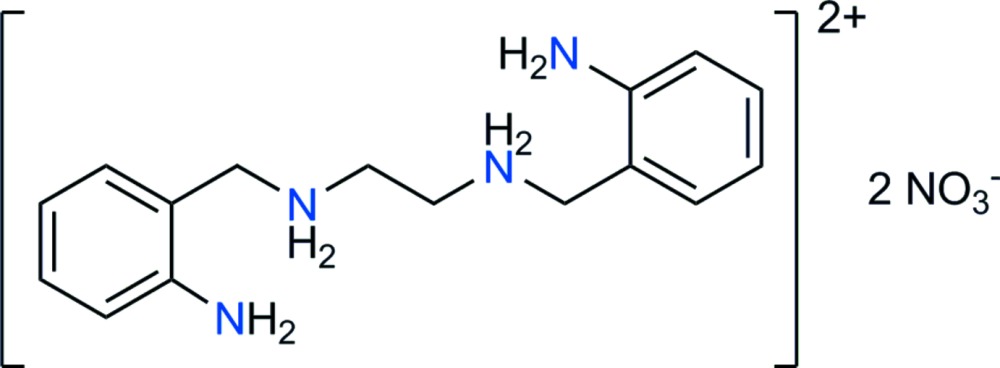


## Experimental

### 

#### Crystal data

C_16_H_24_N_4_^2+^·2NO_3_^−^
*M* *_r_* = 396.41
Orthorhombic, 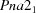

*a* = 11.041 (5) Å
*b* = 5.760 (4) Å
*c* = 30.069 (13) Å
*V* = 1912.1 (18) Å^3^
*Z* = 4
Mo *K*α radiation
μ = 0.11 mm^−1^
*T* = 298 K
0.60 × 0.20 × 0.20 mm

#### Data collection

Siemens P4 diffractometer
4371 measured reflections
2473 independent reflections
1501 reflections with *I* > 2σ(*I*)
*R*_int_ = 0.053
3 standard reflections every 97 reflections intensity decay: 1.5%

#### Refinement

*R*[*F* ^2^ > 2σ(*F* ^2^)] = 0.061
*wR*(*F* ^2^) = 0.180
*S* = 1.61
2473 reflections
278 parameters
13 restraints
H atoms treated by a mixture of independent and constrained refinement
Δρ_max_ = 0.34 e Å^−3^
Δρ_min_ = −0.31 e Å^−3^

### 

Data collection: *XSCANS* (Siemens, 1996 ▶); cell refinement: *XSCANS*; data reduction: *XSCANS*; program(s) used to solve structure: *SHELXS2013* (Sheldrick, 2008 ▶); program(s) used to refine structure: *SHELXL2013* (Sheldrick, 2008 ▶); molecular graphics: *Mercury* (Macrae *et al.*, 2008 ▶); software used to prepare material for publication: *SHELXL2013*.

## Supplementary Material

Crystal structure: contains datablock(s) I, global. DOI: 10.1107/S1600536813027475/rk2415sup1.cif

Structure factors: contains datablock(s) I. DOI: 10.1107/S1600536813027475/rk2415Isup2.hkl

Supplementary material file. DOI: 10.1107/S1600536813027475/rk2415Isup3.cml

Additional supplementary materials:  crystallographic information; 3D view; checkCIF report

## Figures and Tables

**Table 1:** Hydrogen-bond geometry (Å, °)

*D*—H⋯*A*	*D*—H	H⋯*A*	*D*⋯*A*	*D*—H⋯*A*
N9—H9*A*⋯O23^i^	0.94 (4)	1.87 (3)	2.794 (8)	167 (7)
N9—H9*B*⋯O24	0.95 (5)	1.96 (5)	2.879 (7)	164 (5)
N12—H12*A*⋯O27	0.92 (3)	1.88 (3)	2.787 (8)	168 (8)
N12—H12*B*⋯O28^i^	0.91 (5)	1.95 (5)	2.862 (8)	176 (9)
N1—H1*B*⋯O22^ii^	0.90 (7)	2.55 (8)	3.290 (11)	141 (9)
N1—H1*B*⋯O24^ii^	0.90 (7)	2.40 (7)	3.272 (10)	166 (9)
N9—H9*A*⋯O22^i^	0.94 (4)	2.38 (6)	3.050 (8)	128 (5)
N12—H12*A*⋯O26	0.92 (3)	2.36 (5)	3.046 (8)	132 (4)
N12—H12*B*⋯O27^i^	0.91 (5)	2.49 (5)	3.096 (8)	125 (4)
N20—H20*B*⋯O26^iii^	0.91 (7)	2.56 (8)	3.246 (12)	133 (8)
N20—H20*B*⋯O28^iii^	0.91 (7)	2.32 (8)	3.204 (11)	165 (8)
C8—H8*B*⋯O24^ii^	0.97	2.46	3.327 (9)	149
C10—H10*A*⋯O24^ii^	0.97	2.41	3.258 (8)	145
C10—H10*B*⋯O22^iv^	0.97	2.58	3.291 (9)	130
C11—H11*A*⋯O27^i^	0.97	2.56	3.147 (9)	119
C11—H11*A*⋯O26^v^	0.97	2.57	3.285 (9)	131
C11—H11*B*⋯O28^iii^	0.97	2.41	3.249 (9)	145
C13—H13*A*⋯O28^iii^	0.97	2.46	3.315 (10)	147

Symmetry codes: (i) 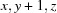; (ii) 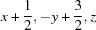; (iii) 
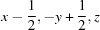
; (iv) 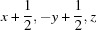; (v) 
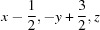
.

**Fig. 1.**
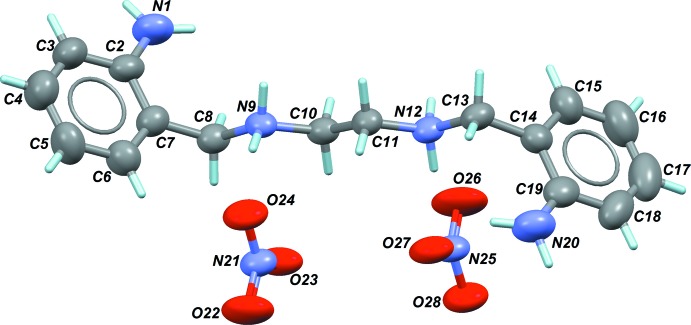
Asymmetric unit of the title compound with the atom numbering scheme. Displacement ellipsoids for non-H atoms are presented at the 50% probability level. H atoms are shown as stick.

**Fig. 2.**
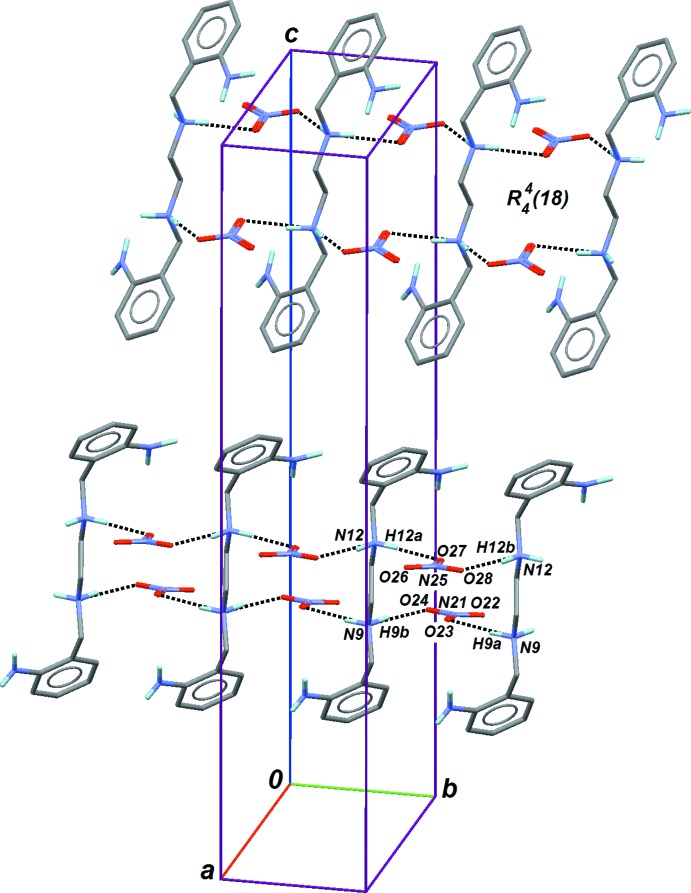
Part of the crystal structure of the title compound, showing N—H···O(nitrate) H bonds as dashed lines. Two supramolecular R(18)-based chains are shown, which are related by the n glide plane perpendicular to [1 0 0]. No significant contacts are observed between chains.

## supplementary crystallographic information

### 1. Comment

The title salt crystallized unexpectedly, when attempting the crystallization of a macrocyclic molecule, resulting from the Schiff condensation between 2,6-diacetylpyridine and *N*,*N*'-bis(2-aminobenzyl)ethane-1,2-diamine (Garza Rodríguez, 2010). The synthesis was carried out *via* a template reaction, using Mn^2+^ as metal center, and analytical data showed that the Mn^2+^ complex was formed, with nitrate as counter ions. However, this compound is almost insoluble in organic solvents, like MeOH, EtOH, acetone and ethyl acetate, impeding the preparation of single crystals. Only slight solubility was obtained in hot acetonitrile. Slow evaporation of MeCN over 3 weeks afforded a mixture of amorphous brown solids and colourless needle-shaped crystals. We assume that the brown solids should be a mixture of manganese oxides, resulting from the hydrolysis of the complex induced by trace amounts of water and dissolved O_2_. Minutes amounts of H_3_O^+^ are then released, which promote the formation, and finally the crystallization of the nitrate salt of the protonated amine.

Title compound (Fig. 1) crystallizes in a non-centrosymmetric space group, with all atoms placed in general positions. However, the dication (C_16_H_24_N_4_)^2+^ presents a non-crystallographic inversion center, with the central aliphatic chain extended in the all-*trans* conformation. This conformation was previously obtained for the same cation crystallized as *p*-toluenesulfonate salt, although in that case, the dication was placed on a crystallographic inversion center (Garza Rodríguez *et al.*, 2011). The free amine, for which the X-ray structure is also known (Rodríguez de Barbarín *et al.*, 2007) has a different solid state conformation, although preserving the centrosymmetric character. For the nitrate salt reported here, departure from centrosymmetry is small, as reflected, for example, by the dihedral angle between benzene rings, limited to 3.3 (6)°.

Nitrate ions positions are determined by the formation of hydrogen bonds with ammonium NH_2_^+^ groups in the cation. All N—H···O angles for these contacts are close to 180°, and H···O separations are in the range 1.87 (3)Å to 1.96 (3)Å. Each independent anion, N21 and N25, bridges two cations related by cell translation in the [0 1 0] direction, forming a *R*_4_^4^(18) ring motif (Etter, 1990; Fig. 2). This arrangement seems actually to be common in crystal structures involving ammonium and nitrate species, and *R*_4_^4^(18) motifs are also formed in salts closely related to the title compound, for example with *N*,*N*'-dibenzylethane-1,2-diammonium (Liu *et al.*, 2007) or *N*,*N*'-bis(4-chlorobenzyl)ethane-1,2-diammonium (Yang *et al.*, 2007). For such salts, the crystal structure is invariably based on edge-fused *R*_4_^4^(18) rings, which afford a one-dimensional linear supramolecular structure. In the case of the title compound, chains run along the short *b* axis, and no significant interchain interactions are observed (Fig. 2).

### 2. Experimental

An amount of 2,6-diacetylpyridine (735 mg, 4.50 mmol) in ethanol (180 ml) was mixed with the templating reagent Mn(NO_3_)_2_·*x*H_2_O (1.130 g) and refluxed for 30 min. Then, *N*,*N*'-bis(2-aminobenzyl)ethane-1,2-diamine (1.302 g, 4.80 mmol, dissolved in 25 ml of ethanol) was slowly added, and the mixture further refluxed for 1 h. The resulting solid was filtered from hot ethanol, washed with cold ethanol, and dried under reduced pressure. A solution of the solid in hot CH_3_CN was left to crystallize for 3 weeks. After this time, only one product was obtained as single crystals, in low yield, which was identified by X-ray diffraction as the title nitrate salt.

### 3. Refinement

H atoms for aromatic CH and methylene CH_2_ groups were placed in idealized positions, and refined with C—H bond lengths fixed to 0.93Å and 0.97Å, respectively, and *U*_iso_(H) = 1.2*U*_eq_(C). Amine and ammonium H atoms were found in a difference map, and refined freely, although the geometry for NH_2_ group was restrained to sensible target values: bond lengths N—H were restrained to 0.90 (2)Å and H···H separations were restrained to 1.54 (3)Å. Isotropic displacement parameters were computed in this case as *U*_iso_(H) = 1.5*U*_eq_(N).

### Crystal data

**Table:**

C_16_H_24_N_4_^2+^·2NO_3_^−^	*D*_x_ = 1.377 Mg m^−^^3^
*M**_r_* = 396.41	Mo *K*α radiation, λ = 0.71073 Å
Orthorhombic, *P**n**a*2_1_	Cell parameters from 75 reflections
*a* = 11.041 (5) Å	θ = 4.9–11.7°
*b* = 5.760 (4) Å	µ = 0.11 mm^−^^1^
*c* = 30.069 (13) Å	*T* = 298 K
*V* = 1912.1 (18) Å^3^	Needle, colourless
*Z* = 4	0.60 × 0.20 × 0.20 mm
*F*(000) = 840	

### Data collection

**Table:**

Siemens P4 diffractometer	*R*_int_ = 0.053
Radiation source: fine-focus sealed tube, FN4	θ_max_ = 25.5°, θ_min_ = 2.7°
Graphite monochromator	*h* = −13→13
2θ/ω–scans	*k* = −3→6
4371 measured reflections	*l* = −36→36
2473 independent reflections	3 standard reflections every 97 reflections
1501 reflections with *I* > 2σ(*I*)	intensity decay: 1.5%

### Refinement

**Table:**

Refinement on *F*^2^	Secondary atom site location: difference Fourier map
Least-squares matrix: full	Hydrogen site location: difference Fourier map
*R*[*F*^2^ > 2σ(*F*^2^)] = 0.061	H atoms treated by a mixture of independent and constrained refinement
*wR*(*F*^2^) = 0.180	*w* = 1/[σ^2^(*F*_o_^2^) + (0.06*P*)^2^] where *P* = (*F*_o_^2^ + 2*F*_c_^2^)/3
*S* = 1.61	(Δ/σ)_max_ < 0.001
2473 reflections	Δρ_max_ = 0.34 e Å^−^^3^
278 parameters	Δρ_min_ = −0.31 e Å^−^^3^
13 restraints	Extinction correction: *SHELXL2013* (Sheldrick, 2008), Fc^*^=kFc[1+0.001xFc^2^λ^3^/sin(2θ)]^-1/4^
0 constraints	Extinction coefficient: 0.010 (2)
Primary atom site location: structure-invariant direct methods	

### Fractional atomic coordinates and isotropic or equivalent isotropic displacement parameters (Å^2^)

**Table:**

	*x*	*y*	*z*	*U*_iso_*/*U*_eq_	
N1	0.9978 (7)	1.1615 (13)	0.1913 (3)	0.083 (2)	
H1A	1.023 (9)	1.261 (14)	0.171 (2)	0.124*	
H1B	1.038 (8)	1.148 (19)	0.2170 (18)	0.124*	
C2	0.9002 (7)	1.0332 (13)	0.1770 (3)	0.0616 (19)	
C3	0.8321 (9)	1.1154 (15)	0.1408 (3)	0.077 (2)	
H3A	0.8541	1.2543	0.1272	0.092*	
C4	0.7348 (9)	0.9958 (16)	0.1251 (3)	0.087 (3)	
H4A	0.6902	1.0542	0.1014	0.104*	
C5	0.7024 (8)	0.7886 (18)	0.1444 (3)	0.080 (2)	
H5A	0.6364	0.7049	0.1339	0.096*	
C6	0.7699 (7)	0.7072 (16)	0.1797 (2)	0.067 (2)	
H6A	0.7485	0.5667	0.1928	0.080*	
C7	0.8672 (7)	0.8267 (13)	0.1961 (2)	0.0564 (18)	
C8	0.9347 (6)	0.7329 (14)	0.2354 (2)	0.0545 (18)	
H8A	0.9438	0.5662	0.2322	0.065*	
H8B	1.0150	0.8010	0.2363	0.065*	
N9	0.8705 (4)	0.7843 (10)	0.27754 (19)	0.0485 (13)	
H9A	0.846 (6)	0.937 (5)	0.284 (3)	0.073*	
H9B	0.795 (4)	0.706 (10)	0.276 (3)	0.073*	
C10	0.9427 (5)	0.7325 (13)	0.3181 (2)	0.0492 (15)	
H10A	1.0124	0.8346	0.3195	0.059*	
H10B	0.9715	0.5734	0.3171	0.059*	
C11	0.8645 (5)	0.7674 (13)	0.3583 (2)	0.0474 (14)	
H11A	0.8372	0.9274	0.3597	0.057*	
H11B	0.7937	0.6679	0.3566	0.057*	
N12	0.9349 (4)	0.7109 (11)	0.39831 (19)	0.0489 (13)	
H12A	0.960 (5)	0.559 (5)	0.398 (3)	0.073*	
H12B	0.999 (4)	0.810 (9)	0.397 (3)	0.073*	
C13	0.8723 (7)	0.7589 (13)	0.4415 (2)	0.0559 (18)	
H13A	0.7905	0.6976	0.4406	0.067*	
H13B	0.8674	0.9252	0.4463	0.067*	
C14	0.9406 (7)	0.6483 (14)	0.4791 (2)	0.056 (2)	
C15	1.0413 (8)	0.7526 (16)	0.4955 (2)	0.073 (2)	
H15A	1.0652	0.8941	0.4835	0.087*	
C16	1.1096 (9)	0.658 (2)	0.5293 (3)	0.093 (3)	
H16A	1.1798	0.7300	0.5392	0.112*	
C17	1.0704 (10)	0.451 (2)	0.5479 (3)	0.094 (3)	
H17A	1.1137	0.3844	0.5711	0.113*	
C18	0.9691 (9)	0.3471 (17)	0.5323 (3)	0.085 (3)	
H18A	0.9439	0.2083	0.5451	0.102*	
C19	0.9016 (7)	0.4411 (14)	0.4977 (3)	0.066 (2)	
N20	0.7978 (8)	0.3323 (14)	0.4831 (3)	0.086 (2)	
H20A	0.786 (10)	0.185 (8)	0.493 (3)	0.128*	
H20B	0.764 (9)	0.363 (17)	0.4563 (19)	0.128*	
N21	0.7142 (5)	0.2743 (11)	0.2797 (2)	0.0550 (14)	
O22	0.6509 (5)	0.1048 (10)	0.2738 (2)	0.093 (2)	
O23	0.8219 (4)	0.2579 (8)	0.28670 (19)	0.0729 (15)	
O24	0.6674 (4)	0.4708 (9)	0.27685 (19)	0.0735 (15)	
N25	1.0910 (5)	0.2211 (10)	0.3951 (2)	0.0556 (14)	
O26	1.1542 (5)	0.3900 (10)	0.3991 (3)	0.099 (2)	
O27	0.9825 (4)	0.2383 (9)	0.38897 (19)	0.0702 (16)	
O28	1.1372 (5)	0.0215 (9)	0.3989 (2)	0.0750 (14)	

### Atomic displacement parameters (Å^2^)

**Table:**

	*U*^11^	*U*^22^	*U*^33^	*U*^12^	*U*^13^	*U*^23^
N1	0.097 (5)	0.055 (5)	0.095 (5)	−0.021 (4)	−0.007 (4)	0.007 (4)
C2	0.077 (5)	0.041 (4)	0.066 (4)	−0.003 (4)	0.006 (4)	0.001 (4)
C3	0.110 (7)	0.055 (5)	0.066 (4)	−0.006 (5)	−0.004 (5)	0.010 (4)
C4	0.104 (7)	0.085 (7)	0.071 (5)	−0.002 (7)	−0.015 (5)	0.011 (5)
C5	0.074 (5)	0.100 (7)	0.067 (4)	−0.016 (5)	−0.012 (4)	−0.002 (5)
C6	0.063 (4)	0.069 (6)	0.068 (5)	−0.007 (4)	−0.006 (4)	0.000 (4)
C7	0.061 (4)	0.051 (5)	0.057 (4)	0.001 (4)	0.005 (3)	0.006 (4)
C8	0.060 (4)	0.047 (4)	0.057 (4)	0.003 (4)	0.009 (3)	0.002 (4)
N9	0.051 (3)	0.036 (3)	0.058 (3)	0.005 (3)	−0.006 (3)	−0.002 (3)
C10	0.048 (4)	0.039 (4)	0.061 (4)	0.000 (3)	−0.003 (3)	0.002 (4)
C11	0.048 (3)	0.040 (4)	0.054 (3)	0.003 (3)	−0.005 (3)	0.003 (4)
N12	0.053 (3)	0.037 (3)	0.056 (3)	0.006 (3)	0.003 (3)	0.003 (3)
C13	0.064 (4)	0.042 (4)	0.062 (4)	0.001 (4)	0.005 (3)	0.008 (4)
C14	0.066 (5)	0.047 (5)	0.054 (4)	0.000 (4)	0.001 (3)	−0.004 (4)
C15	0.080 (5)	0.079 (6)	0.059 (4)	−0.002 (5)	−0.005 (4)	−0.007 (5)
C16	0.089 (6)	0.119 (9)	0.071 (5)	−0.003 (6)	−0.009 (5)	−0.016 (6)
C17	0.102 (7)	0.120 (9)	0.060 (5)	0.040 (7)	−0.005 (5)	−0.006 (6)
C18	0.114 (7)	0.077 (7)	0.062 (5)	0.026 (6)	0.015 (5)	0.014 (5)
C19	0.082 (5)	0.050 (5)	0.065 (4)	0.010 (4)	0.012 (4)	−0.002 (4)
N20	0.096 (6)	0.057 (5)	0.103 (5)	−0.015 (5)	0.002 (5)	0.009 (4)
N21	0.050 (3)	0.041 (4)	0.074 (3)	0.005 (3)	0.001 (3)	0.005 (4)
O22	0.077 (4)	0.049 (3)	0.151 (6)	−0.016 (3)	−0.005 (4)	−0.003 (4)
O23	0.051 (3)	0.043 (3)	0.125 (4)	0.000 (3)	−0.014 (3)	−0.004 (4)
O24	0.063 (3)	0.047 (3)	0.111 (4)	0.015 (3)	0.002 (3)	0.004 (3)
N25	0.054 (3)	0.037 (4)	0.075 (3)	0.005 (3)	0.003 (3)	0.001 (4)
O26	0.074 (4)	0.050 (4)	0.174 (6)	−0.023 (3)	−0.001 (4)	0.005 (5)
O27	0.055 (3)	0.039 (3)	0.116 (4)	0.004 (3)	−0.013 (3)	0.008 (4)
O28	0.067 (3)	0.045 (3)	0.113 (4)	0.014 (3)	0.001 (3)	0.005 (3)

### Geometric parameters (Å, º)

**Table:**

N1—C2	1.376 (10)	N12—C13	1.498 (9)
N1—H1A	0.89 (2)	N12—H12A	0.92 (2)
N1—H1B	0.90 (2)	N12—H12B	0.91 (2)
C2—C7	1.371 (9)	C13—C14	1.500 (10)
C2—C3	1.405 (11)	C13—H13A	0.9700
C3—C4	1.360 (12)	C13—H13B	0.9700
C3—H3A	0.9300	C14—C15	1.356 (11)
C4—C5	1.374 (12)	C14—C19	1.386 (11)
C4—H4A	0.9300	C15—C16	1.378 (12)
C5—C6	1.379 (11)	C15—H15A	0.9300
C5—H5A	0.9300	C16—C17	1.382 (15)
C6—C7	1.368 (10)	C16—H16A	0.9300
C6—H6A	0.9300	C17—C18	1.354 (12)
C7—C8	1.497 (10)	C17—H17A	0.9300
C8—N9	1.482 (8)	C18—C19	1.389 (12)
C8—H8A	0.9700	C18—H18A	0.9300
C8—H8B	0.9700	C19—N20	1.378 (11)
N9—C10	1.486 (8)	N20—H20A	0.91 (2)
N9—H9A	0.94 (2)	N20—H20B	0.91 (2)
N9—H9B	0.95 (2)	N21—O23	1.212 (6)
C10—C11	1.501 (8)	N21—O22	1.213 (7)
C10—H10A	0.9700	N21—O24	1.247 (7)
C10—H10B	0.9700	N25—O26	1.203 (7)
C11—N12	1.468 (9)	N25—O27	1.215 (7)
C11—H11A	0.9700	N25—O28	1.263 (7)
C11—H11B	0.9700		
			
C2—N1—H1A	112 (6)	N12—C11—H11B	109.9
C2—N1—H1B	128 (6)	C10—C11—H11B	109.9
H1A—N1—H1B	120 (5)	H11A—C11—H11B	108.3
C7—C2—N1	122.8 (7)	C11—N12—C13	115.1 (5)
C7—C2—C3	118.4 (7)	C11—N12—H12A	112 (5)
N1—C2—C3	118.8 (8)	C13—N12—H12A	109 (5)
C4—C3—C2	121.3 (8)	C11—N12—H12B	104 (5)
C4—C3—H3A	119.3	C13—N12—H12B	106 (5)
C2—C3—H3A	119.3	H12A—N12—H12B	111 (4)
C3—C4—C5	120.0 (8)	N12—C13—C14	110.1 (6)
C3—C4—H4A	120.0	N12—C13—H13A	109.6
C5—C4—H4A	120.0	C14—C13—H13A	109.6
C4—C5—C6	118.7 (9)	N12—C13—H13B	109.6
C4—C5—H5A	120.7	C14—C13—H13B	109.6
C6—C5—H5A	120.7	H13A—C13—H13B	108.2
C7—C6—C5	122.0 (9)	C15—C14—C19	119.3 (8)
C7—C6—H6A	119.0	C15—C14—C13	119.9 (7)
C5—C6—H6A	119.0	C19—C14—C13	120.8 (7)
C6—C7—C2	119.6 (7)	C14—C15—C16	122.7 (9)
C6—C7—C8	119.6 (7)	C14—C15—H15A	118.6
C2—C7—C8	120.8 (7)	C16—C15—H15A	118.6
N9—C8—C7	111.4 (5)	C15—C16—C17	117.9 (10)
N9—C8—H8A	109.3	C15—C16—H16A	121.1
C7—C8—H8A	109.3	C17—C16—H16A	121.1
N9—C8—H8B	109.3	C18—C17—C16	120.0 (9)
C7—C8—H8B	109.3	C18—C17—H17A	120.0
H8A—C8—H8B	108.0	C16—C17—H17A	120.0
C8—N9—C10	113.9 (5)	C17—C18—C19	121.9 (9)
C8—N9—H9A	120 (5)	C17—C18—H18A	119.0
C10—N9—H9A	100 (5)	C19—C18—H18A	119.0
C8—N9—H9B	107 (5)	N20—C19—C14	121.5 (8)
C10—N9—H9B	114 (5)	N20—C19—C18	120.4 (9)
H9A—N9—H9B	102 (4)	C14—C19—C18	118.1 (8)
N9—C10—C11	109.0 (4)	C19—N20—H20A	116 (6)
N9—C10—H10A	109.9	C19—N20—H20B	122 (6)
C11—C10—H10A	109.9	H20A—N20—H20B	115 (5)
N9—C10—H10B	109.9	O23—N21—O22	121.8 (6)
C11—C10—H10B	109.9	O23—N21—O24	119.3 (6)
H10A—C10—H10B	108.3	O22—N21—O24	118.8 (6)
N12—C11—C10	109.0 (4)	O26—N25—O27	121.4 (6)
N12—C11—H11A	109.9	O26—N25—O28	119.5 (6)
C10—C11—H11A	109.9	O27—N25—O28	119.1 (6)
			
C7—C2—C3—C4	0.8 (12)	C10—C11—N12—C13	174.5 (5)
N1—C2—C3—C4	179.9 (9)	C11—N12—C13—C14	167.8 (7)
C2—C3—C4—C5	−1.0 (14)	N12—C13—C14—C15	79.3 (9)
C3—C4—C5—C6	0.4 (14)	N12—C13—C14—C19	−101.3 (7)
C4—C5—C6—C7	0.2 (13)	C19—C14—C15—C16	2.1 (12)
C5—C6—C7—C2	−0.4 (12)	C13—C14—C15—C16	−178.5 (7)
C5—C6—C7—C8	178.2 (7)	C14—C15—C16—C17	−2.2 (13)
N1—C2—C7—C6	−179.2 (7)	C15—C16—C17—C18	1.2 (13)
C3—C2—C7—C6	−0.1 (11)	C16—C17—C18—C19	0.0 (13)
N1—C2—C7—C8	2.2 (11)	C15—C14—C19—N20	177.9 (8)
C3—C2—C7—C8	−178.7 (7)	C13—C14—C19—N20	−1.6 (11)
C6—C7—C8—N9	−79.6 (9)	C15—C14—C19—C18	−0.8 (11)
C2—C7—C8—N9	99.1 (7)	C13—C14—C19—C18	179.8 (7)
C7—C8—N9—C10	−169.9 (6)	C17—C18—C19—N20	−178.9 (8)
C8—N9—C10—C11	−174.2 (6)	C17—C18—C19—C14	−0.2 (12)
N9—C10—C11—N12	178.7 (7)		

### Hydrogen-bond geometry (Å, º)

**Table:**

*D*—H···*A*	*D*—H	H···*A*	*D*···*A*	*D*—H···*A*
N9—H9*A*···O23^i^	0.94 (4)	1.87 (3)	2.794 (8)	167 (7)
N9—H9*B*···O24	0.95 (5)	1.96 (5)	2.879 (7)	164 (5)
N12—H12*A*···O27	0.92 (3)	1.88 (3)	2.787 (8)	168 (8)
N12—H12*B*···O28^i^	0.91 (5)	1.95 (5)	2.862 (8)	176 (9)
N1—H1*B*···O22^ii^	0.90 (7)	2.55 (8)	3.290 (11)	141 (9)
N1—H1*B*···O24^ii^	0.90 (7)	2.40 (7)	3.272 (10)	166 (9)
N9—H9*A*···O22^i^	0.94 (4)	2.38 (6)	3.050 (8)	128 (5)
N12—H12*A*···O26	0.92 (3)	2.36 (5)	3.046 (8)	132 (4)
N12—H12*B*···O27^i^	0.91 (5)	2.49 (5)	3.096 (8)	125 (4)
N20—H20*B*···O26^iii^	0.91 (7)	2.56 (8)	3.246 (12)	133 (8)
N20—H20*B*···O28^iii^	0.91 (7)	2.32 (8)	3.204 (11)	165 (8)
C8—H8*B*···O24^ii^	0.97	2.46	3.327 (9)	149
C10—H10*A*···O24^ii^	0.97	2.41	3.258 (8)	145
C10—H10*B*···O22^iv^	0.97	2.58	3.291 (9)	130
C11—H11*A*···O27^i^	0.97	2.56	3.147 (9)	119
C11—H11*A*···O26^v^	0.97	2.57	3.285 (9)	131
C11—H11*B*···O28^iii^	0.97	2.41	3.249 (9)	145
C13—H13*A*···O28^iii^	0.97	2.46	3.315 (10)	147

Symmetry codes: (i) *x*, *y*+1, *z*; (ii) *x*+1/2, −*y*+3/2, *z*; (iii) *x*−1/2, −*y*+1/2, *z*; (iv) *x*+1/2, −*y*+1/2, *z*; (v) *x*−1/2, −*y*+3/2, *z*.
